# CARM1 and BAF155: an example of how chromatin remodeling factors can be relocalized and contribute to cancer

**DOI:** 10.1186/bcr3657

**Published:** 2014-05-19

**Authors:** Olafur A Stefansson, Manel Esteller

**Affiliations:** 1 Cancer Epigenetics and Biology Program (PEBC), Bellvitge Biomedical Research Institute (IDIBELL), Av. Gran Via de L’Hospitalet 199-203, L’Hospitalet del Llobregat, Barcelona 08908, Catalonia, Spain; 2 Cancer Research Laboratory, Faculty of Medicine, University of Iceland, Sæmundargötu 2, 101, Reykjavík, Iceland; 3 Department of Physiological Sciences II, School of Medicine, University of Barcelona, Barcelona 08908, Catalonia, Spain; 4 Institució Catalana de Recerca i Estudis Avançats (ICREA), Barcelona 08010, Catalonia, Spain

## Abstract

In a recent article, Wang and colleagues reported the discovery of a mechanism by which CARM1 regulates the genomic localization of BAF155 (a SWI/SNF subunit involved in chromatin remodeling) through post-translational methylation at R1064 arginine residues. This modification leads to the relocalization of BAF155-containing SWI/SNF complexes to regions containing genes involved in the Myc oncogenic pathway. The results presented are evidence that these interactions constitute a mechanism by which the BAF155 chromatin remodeling factor contributes to cancer.

## Background

A consistent finding across various cancer genome sequencing projects is the identification of recurrent mutations in genes known to be functionally involved in processing histone marks or controlling chromatin dynamics. These mutations are collectively referred to as epigenetic genes. As an example, recurrent mutations in breast cancer are found in chromatin remodeling factors such as ARID1A, ARID1B and SMARCD1 [1-4]. These observations reinforce the link between deregulated chromatin remodeling processes and cancer development, as research indicated as far back as the late 1990s [5]. But how? What is the mechanism? Mechanistic insight has been lacking and there is very little direct information to show how deregulated chromatin remodeling, whether through acquired mutations in genes encoding remodeling factors or not, can contribute to cancer [6]. Wang and colleagues recently carried out elaborate experiments that enabled them to identify BAF155 (a subunit of the SWI/SNF complex) as a substrate for CARM1 methyltransferase and to provide insight into the mechanism by which this factor can lead to or enhance the process of cancer origin development and progression [7].

## CARM1-mediated methylation of BAF155 at arginine residue R1064

The results presented by Wang and colleagues bring into sharp focus the limitations of RNA interference compared with the advantages of recently developed genome editing techniques for studying the biological activities of enzymes *in vivo*. Using an experimental system for monitoring the dynamics of arginine methylation, and through short hairpin RNA-mediated knockdown of CARM1 mRNA transcripts (90% reduction) in MCF7 breast cancer cell lines, the authors demonstrate that the RNA interference technique is insufficient to reduce CARM1 methyltransferase functionality. Even very limited expression levels of CARM1 were shown to be sufficient to sustain its methyltransferase activity. Using the zinc finger nuclease technique, CARM1 knockout clones were generated for the MCF7 and MDA-MB-231 breast cancer models and the HEK293T kidney cell line and were validated as CARM1 dysfunctional.

By carrying out immunoprecipitation and mass spectrometry, Wang and colleagues identified BAF155 (also known as SMARCC1) as a substrate of CARM1 methyltransferase activity [7]. The authors then demonstrated that methylation of BAF155 at arginine residue R1064 affects the colony-formation capacity of MCF7 breast cancer cells and that this modification is entirely dependent on CARM1. Chromatin immunoprecipitation and high-throughput DNA sequencing of BAF155 further showed that BAF155 arginine methylation dramatically affects its genomic location. Methyl-BAF155 was found to be enriched at genes involved in the c-Myc pathway – well known for its link to carcinogenesis – and to be a potential marker for clinical applications in cancer diagnosis and prognosis.

## The clinical value of deregulated chromatin remodeling factors in cancer: a novel therapeutic approach?

In the past few years the field of cancer epigenetics has attracted considerable attention because of its potential in the area of personalized medicine [8]. The results of Wang and colleagues contribute to this purpose by suggesting a potential therapeutic approach for targeting the regulators of chromatin remodeling factors; that is, CARM1 methyltransferase inhibition by small-molecule drug compounds. This would be predicted to inhibit relocalization of BAF155 and thereby abolish its ability to promote the expression of Myc-pathway oncogenes. This therapeutic option could be useful in the context of lowering the risk of disease relapse or, equally relevant, as a cancer-preventative strategy in high-risk groups (for example, in BRCA1 or BRCA2 mutation carriers). RNA interference knockdown experiments revealed CARM1 be a difficult target, however, so it might be impossible to inhibit the methyltransferase effectively at clinically achievable doses. Nevertheless, it is intriguing that, by targeting post-translational modifiers of chromatin remodeling factors, we could influence whether they occupy genes that confer either oncogenic or tumor-suppressive properties.

Several cancer-associated chromatin remodeling factors other than BAF155 have already been identified in breast cancer; for example, ARID1A, ARID1B, SMARCD1, PBRM1, BAF60A and BRG1. Exciting new evidence is emerging from the discovery that ARID1A-mutant cancer cells are highly vulnerable to loss of ARID1B expression [9], and similarly for BRG1/SMARCA4-mutated cancers to the loss of BRM/SMARCA2 [10]. Another notable example in this context includes the development of LSD1 drug inhibitors, since this gene is part of the NuRD-chromatin remodeling complex [11].

In conclusion, the discoveries made by Wang and colleagues are an important source of insight as they not only provide a mechanistic explanation of how deregulated chromatin remodeling factors can contribute to cancer, but also hint at therapeutic potentials that deserve further attention in preclinical research.

## Competing interests

The authors declare that they have no competing interests.
